# High-Resolution Mapping of *Barley mild mosaic virus* Resistance Gene *rym15*

**DOI:** 10.3389/fpls.2022.908170

**Published:** 2022-06-02

**Authors:** Yaping Wang, Antje Habekuß, Murukarthick Jayakodi, Martin Mascher, Rod J. Snowdon, Andreas Stahl, Janina Fuß, Frank Ordon, Dragan Perovic

**Affiliations:** ^1^Institute for Resistance Research and Stress Tolerance, Federal Research Centre for Cultivated Plants, Julius Kuehn-Institute (JKI), Quedlinburg, Germany; ^2^Department of Genebank, Leibniz Institute of Plant Genetics and Crop Plant Research (IPK) Gatersleben, Seeland, Germany; ^3^German Centre for Integrative Biodiversity Research (iDiv) Halle-Jena-Leipzig, Leipzig, Germany; ^4^Department of Plant Breeding, IFZ Research Centre for Biosystems, Land Use and Nutrition, Justus Liebig University, Giessen, Germany; ^5^Institute for Clinical Molecular Biology, Competence Centre for Genomic Analysis (CCGA), Kiel University, Kiel, Germany

**Keywords:** barley, BaMMV resistance, high-resolution mapping, *rym15*, candidate gene

## Abstract

*Barley yellow mosaic virus* (BaYMV) and *Barley mild mosaic virus* (BaMMV), which are transmitted by the soil-borne plasmodiophorid *Polymyxa graminis*, cause high yield losses in barley. In previous studies, the recessive BaMMV resistance gene *rym15*, derived from the Japanese landrace Chikurin Ibaraki 1, was mapped on chromosome 6HS of *Hordeum vulgare*. In this study, 423 F_4_ segmental recombinant inbred lines (RILs) were developed from crosses of Chikurin Ibaraki 1 with two BaMMV-susceptible cultivars, Igri (139 RILs) and Uschi (284 RILs). A set of 32 competitive allele-specific PCR (KASP) assays, designed using single nucleotide polymorphisms (SNPs) from the barley 50 K Illumina Infinium iSelect SNP chip, genotyping by sequencing (GBS) and whole-genome sequencing (WGS), was used as a backbone for construction of two high-resolution maps. Using this approach, the target locus was narrowed down to 0.161 cM and 0.036 cM in the Igri × Chikurin Ibaraki 1 (I × C) and Chikurin Ibaraki 1 × Uschi (C × U) populations, respectively. Corresponding physical intervals of 11.3 Mbp and 0.281 Mbp were calculated for I × C and C × U, respectively, according to the Morex v3 genome sequence. In the 0.281 Mbp target region, six high confidence (HC) and two low confidence (LC) genes were identified. Genome assemblies of BaMMV-susceptible cultivars Igri and Golden Promise from the barley pan-genome, and a HiFi assembly of Chikurin Ibaraki 1 together with re-sequencing data for the six HC and two LC genes in susceptible parental cultivar Uschi revealed functional SNPs between resistant and susceptible genotypes only in two of the HC genes. These SNPs are the most promising candidates for the development of functional markers and the two genes represent promising candidates for functional analysis.

## Introduction

Barley (*Hordeum vulgare* ssp. *vulgare*), the fourth most cultivated cereal in the world (FAOSTAT, 2022), is mainly used for animal feed and malting. The soil-borne barley yellow mosaic disease, caused by *Barley yellow mosaic virus* (BaYMV) and *Barley mild mosaic virus* (BaMMV), significantly affects the yield of winter barley in large parts of Europe and East Asia (Kühne, 2009). Due to transmission of BaMMV and BaYMV *via* the soil-borne plasmodiophorid *Polymyxa graminis* (Adams et al., 1988; Kanyuka et al., 2003), it is of prime importance to improve the genetic resistance in modern cultivars to ensure winter barley cultivation despite the increasing frequency of infested fields.

A total of 22 resistance genes against BaYMV and/or BaMMV were reported up to now, of which the two recessive genes *rym1/11* and *rym4*/*5* have been the predominant sources of breeding for commercial BaMMV/BaYMV resistant barley cultivars (Jiang et al., 2020). However, a predominant European isolate BaYMV-2 became virulent on *rym4*-carrying barley varieties (Kühne et al., 2003; Rolland et al., 2017). Another widespread BaYMV-2 resistance gene *rym5* is overcome by the European isolates BaMMV-Teik and BaMMV-SIL (Kanyuka et al., 2004; Habekuß et al., 2007), the Japanese isolate BaYMV-III (Nishigawa et al., 2008) and isolates of BaMMV in France (Rolland et al., 2017). In addition, in China, BaYMV isolates BaYMV-CN_NY and BaYMV-CN_YZ were virulent to *rym4*, and *rym5* was overcome by BaYMV isolates BaYMV-CN_DZ and BaYMV-CN_NY, as well as BaMMV isolates BaMMV-CN_NY and BaMMV-CN_YZ. Remarkably, the isolate BaYMV-CN_NY was also virulent to accessions, which carried *rym1*/*11* and *rym5* (Jiang et al., 2022). Thus, it is critical to search for alternative BaMMV/BaYMV resistance resources and identify diagnostic markers for marker-assisted selection.

During recent decades, in addition to SNP arrays (Bayer et al., 2017), next-generation sequencing (NGS) technologies have been widely applied in plant breeding. For instance, using NGS technology, cost-effective genotyping-by-sequencing (GBS) approaches have been developed and widely used in barley genetic studies (Poland et al., 2012). SNPs assayed with high-density SNP arrays and GBS enable navigation between genetic maps and physical genome positions. Using both kinds of markers in tandem can be advantageous because polymorphisms of GBS-derived SNPs and SNPs included in arrays tend to target complementary haplotypes or genome regions (Darrier et al., 2019; Negro et al., 2019). Furthermore, GBS-derived SNPs have more power to detect rare alleles in diverse germplasm collections, while SNP arrays are prone to ascertainment bias. On the other hand, array-derived SNPs have the advantage of highly robust calling of alleles at the same SNPs across multiple populations (Darrier et al., 2019).

Third-generation sequencing technologies, such as Pacific Biosciences (PacBio) and Oxford Nanopore Technologies, operate on different principles (Eid et al., 2009; Jain et al., 2015). Compared to the short-read approaches, the assembly data obtained by using long-read sequencing methods can provide more information regarding variants residing in the repeat-rich intergenic space or copy-number variants at complex loci (Mascher et al., 2021). However, until very recently, error rates of both sequencing platforms were significantly higher than short-read NGS methods (Hu et al., 2021). Depending on the DNA fragment length and quality, Oxford Nanopore Technologies MinION/GridION can provide reads longer than 1 Mb, with read accuracy of 87–98% and reads for an N50 of 10–60 kb, and the newest PacBio sequencing improvement Sequel 2 can generate high-fidelity (HiFi) reads up to 20 kb with more than 99% accuracy with N50 of 10–20 kb using the single-molecule circular consensus sequence technology (Wenger et al., 2019; Logsdon et al., 2020; Miga et al., 2020).

Recently, a barley pan-genome was assembled comprising 19 cultivated accessions and one wild barley (Jayakodi et al., 2020). Furthermore, the updated barley reference genome Morex v3 was released by the use of accurate circular consensus long-read sequencing, and a set of 35,827 high confidence (HC) and 45,860 low confidence (LC) genes was identified (Mascher, 2020; Mascher et al., 2021). The availability of those online resources facilitates the study of the genome and its relationship with target traits in barley. For the present study, the assembly of our susceptible parental line Igri is of particular relevance as a sequence resource for narrowing down and annotating the *rym15* target region.

In the past 20 years, map-based cloning turned out to be efficient for the isolation of candidate genes for important traits (Jaganathan et al., 2020). Up to now, two BaMMV/BaYMV resistance loci were cloned through map-based cloning: *rym4*, *rym5*, and *rym_HOR3298_*, as allelic variants of the eukaryotic translation initiation factor 4E (*eIF4E*; Kanyuka et al., 2005; Stein et al., 2005; Shi et al., 2019), and *rym1/11* encoding a protein disulfide isomerase like 5–1 (*PDIL5-1*; Yang et al., 2014). The updated and improved genomic resources for barley have simplified marker saturation and accelerated gene isolation (Perovic et al., 2018). The availability of public reference genome assemblies and low-cost, high throughput sequencing platforms, which can generate millions of polymorphisms for genetic mapping, provide a great opportunity for genetic mapping studies (Jaganathan et al., 2020).

Chikurin Ibaraki 1 is susceptible to BaYMV in Japan (Ukai and Yamashita, 1980). Interestingly, this Japanese cultivar was found to be resistant to three European strains, that is, BaMMV, BaYMV-1, and BaYMV-2 (Götz and Friedt, 1993; Lapierre and Signoret, 2004). The first genetic mapping of the Chikurin Ibaraki 1 derived BaMMV resistance locus *rym15* revealed that it is inherited recessively and located on chromosome 6HS (Le Gouis et al., 2004). In a previous publication (Wang et al., 2021), two medium-resolution maps were constructed by using a set of 180 (I × C) and 342 (C × U) F_2_ plants. In this publication mapping was done by the use of six SSR markers and eight KASP markers (*rym15*_1 to *rym15*_17) that were developed based on a 50 K Illumina Infinium iSelect screen of three parental lines and phenotyping of corresponding F_2_-F_3_ families, the gene was fixed between KASP markers *rym15*_1 and *rym15*_8 in an interval around 137 Mb according to the barley reference assembly Morex v2 (Wang et al., 2021). Based on this information, in a current study, two high-resolution mapping populations comprising 2,218 (I × C) and 5,870 (C × U) F_2_ plants were developed and corresponding F_4_ segmental RILs were phenotyped using the BaMMV-ASL isolate, the present study aimed to (1) construct a high-resolution mapping population of *rym15*, (2) narrow down the target region, and (3) predict potential candidate genes for BaMMV resistance gene *rym15*.

## Materials and Methods

### Plant Material and Construction of the High-Resolution Mapping Populations

To construct high-resolution mapping populations for *rym15*, two segregating F_2_ populations comprising 2,218 and 5,870 F_2_ plants were produced based on the crosses between the resistant cultivar Chikurin Ibaraki 1 and the susceptible cultivars Igri and Uschi, respectively. DNA of F_2_ plants was extracted at the two-leaf stage using the efficient 96-sample multiplex DNA extraction protocol described by Milner et al. (2019). All F_2_ plants were analyzed using the co-dominant flanking markers *rym15*_1 and *rym15*_8 which we identified in a previous study (Wang et al., 2021). Those F_2_ plants carrying a recombination event within the target interval were self-pollinated and selfed seeds were harvested. For each recombinant F_2_ plant, a set of 12 seeds was sown in 96 Quick pot trays (8 × 12). DNA of F_3_ plants was extracted as described above and subsequently analyzed with the same markers, that is, *rym15*_1 and *rym15*_8, in order to identify segmental homozygous recombinants. Homozygous recombinant F_3_ plants were selfed and corresponding F_4_ plants were subsequently used for the construction of a high-resolution mapping population. By this approach, two high-resolution mapping populations of 139 (I × C) and 284 (C × U) F_4_ segmental RILs were developed and subsequently used for resistance testing (Table 1).

**Table 1:** Screening of *F*_2_ plants for the construction of *rym15* high-resolution mapping populations.

Crosses	Number of analyzed *F*_2_ plants	Number of recombinant *F*_2_ plants	Recombination frequency	Number of segmental RILs (F_4_)		*χ* ^2^ 1:1 (*df* = 1, *p* > 0.05)
				Resistant	Susceptible	
Igri × Chikurin Ibaraki 1	2,174	162	3.72%	67	72	0.18 (*P* = 0.6714)
Chikurin Ibaraki 1 × Uschi	5,728	288	2.51%	140	144	0.056 (*P* = 0.8129)

### Resistance Test

All 423 RILs were mechanically inoculated with a predominant isolate BaMMV-ASL (derived from Aschersleben, Germany) under controlled growth chamber conditions according to Perovic et al. (2014). A set of 6 plants per segmental RIL were sown randomly in 60 Quick pot trays (6 × 10). In each tray, a set of 6 plants of cultivar Maris Otter was used as positive control, and three plants of the resistant parent Chikurin Ibaraki 1 and three plants of the susceptible parent Igri or Uschi were sown. Five to six weeks after the first inoculation, the mosaic symptoms on the plants were estimated visually and the double antibody sandwich enzyme-linked immunosorbent assay (DAS-ELISA) was carried out according to Clark and Adams (1977), using polyclonal antibodies and conjugate IgG (Loewe Biochemica, Sauerlach, Cat. No.07006S). The virus titer was estimated *via* extinction at 405 nm using a Dynatech MR 5000 microtiter-plate reader at 45 min and 90 min after addition of p-Nitrophenyl Phosphate (PNPP). Plants with an extinction E405 > 0.10 were qualitatively scored as susceptible. Segregation of resistant and susceptible F_4_ RILs was analyzed using the chi-square tests for goodness of fit to the expected segregation ratios (1r:1 s).

### GBS Library Construction, Sequencing, and Data Analysis

Genomic DNA of the parental lines (Chikurin Ibaraki 1, Igri, and Uschi) was extracted using the CTAB (cetyl trimethylammonium bromide) method according to Stein et al. (2001) and digested with *PstI* and *MspI* (New England Biolabs) according to Wendler et al. (2014). GBS libraries were loaded on 2% Ultra PureTM Agarose Gel from Invitrogen stained with SYBRGold. Size selection from 250 bp to 600 bp was performed visually and gel extraction of cut gel pieces was performed using MinElute Gel Extraction Kit from Qiagen. The GBS libraries were sequenced in loading concentration of 10pM on Illumina® MiSeq™ (Illumina, San Diego, United States) with 150 cycles, single-end reads, using a custom sequencing primer. Sequence data were analyzed using a Galaxy web server (Giardine et al., 2005; Blankenberg et al., 2010; Goecks et al., 2010). The sequencing reads were trimmed by using the tool Trim Galore (version 0.4.0) with a quality threshold of 30 to remove the low-quality reads and also the reads shorter than 50 bp. Alignment was performed against the genome assembly Morex v3 (Mascher, 2020) by using the trimmed sequencing reads of three parental lines. This step was conducted using BWA-MEM (version 0.7.17; Li, 2013) with default parameters. SNP calling was performed using MPileup version 1.8 (Li and Durbin, 2009) and the polymorphisms between resistant (Chikurin Ibaraki 1) and susceptible (Igri and Uschi) parental lines were filtered in the resulting VCF file (Danecek et al., 2011). Variant sites were retained in case they presented a minimum SNP quality score of 40, minimum genotype quality of 5, and minimum number of homozygous/heterozygous reads covering a position per sample of 2/4.

### Whole-Genome Re-Sequencing of Chikurin Ibaraki 1 and Data Analysis

In order to obtain the whole-genome sequencing data of resistance donor line Chikurin Ibaraki 1, a seed bulk of Chikurin Ibaraki 1 was grown for 7 days and dark treated for 48 h (INRA-CNRGV Plant Genomic Center, Toulouse, France). High molecular weight (HMW) DNA was isolated using a Qiagen G-100 DNA extraction kit following the manufacturer’s protocol (https://www.qiagen.com/us/products/discovery-and-translational-research/dna-rna-purification/dna-purification/genomic-dna/qiagen-genomic-tips/). The DNA was quantified on a QBit (Invitrogen) and the quality was checked by using NanoDrop One (Thermo Scientific) according to the A260/A280 and A260/A230 ratios. The fragment size estimation was conducted by using the FEMTO pulse (Agilent). Subsequently, lyophilized DNA samples were used for PacBio SMRT sequencing (Center for Genomic Analysis, University of Kiel). Library preparation was conducted using the HiFi SMRTbell Express 2.0 kit (Pacific Biosciences, Menlo Park, USA) including BluePippin (Sage Science Inc., Beverly Massachusetts) size selection with a lower cutoff of 10 kb. Sequencing was performed on the Sequel II instrument on 6 SMRTcell 8 M, movie time of 30 h (Pacific Biosciences, Menlo Park, USA). PacBio HiFi data was assembled with the HiFi read assembler hifiasm (Cheng et al., 2021). The HiFi reads were deposited under project ID PRJEB50079 at the European Nucleotide Archive (ENA).

### Marker Saturation

Genomic DNA of the constructed 423 segmental homozygous F_4_ RILs was extracted using the CTAB method according to Stein et al. (2001). DNA samples of RILs were adjusted to a final concentration of 20 ng/μl and subsequently used for marker saturation.

Based on the physical position of the previous flanking markers *rym15*_1 and *rym15*_8, a set of 28 SNPs derived from the 50 K Illumina Infinium iSelect SNP chip (8 SNPs), GBS (8 SNPs) and assembly data (12 SNPs) located in the target interval was converted to KASP markers using BatchPrimer3 and PolyMarker (You et al., 2008; Ramirez-Gonzalez et al., 2015) algorithms. Furthermore, another two KASP markers located between markers *rym15*_1 and *rym15*_8 were selected from a previous study (Wang et al., 2021; Supplementary Table S1).

The high-resolution mapping populations derived from crosses I × C and C × U were genotyped using 32 and 29 KASP markers, respectively (Supplementary Table S1). PCR amplification was conducted in a 5 μl reaction volume consisting of 2.5 μl PACE™ (PCR Allele Competitive Extension) Genotyping Master Mix (Part. No.001–0002, 3CR Bioscience), 0.08 μl of each allele-specific primer 1 and allele-specific primer 2 (10.0 pmol/μl), 0.2 μl common primer (10.0 pmol/μl) and 2.2 μl template DNA (20 ng/μl). For KASP analysis, DNA was amplified in the CFX96 Touch Real-Time PCR Detection System (Bio-Rad, Hercules, CA, USA) with the following conditions: 94°C for 15 min; followed by PCR with 9 cycles of 20 s at 94°C, 1 min at 61°C; and then 25 cycles with 20 s at 94°C, 1 min at 55°C, and a final cool down at 37°C for 1 min. If necessary, a re-cycle with the following conditions was performed: 94°C for 3 min; followed by PCR with 9 cycles of 20 s at 94°C, 1 min at 57°C and a final cool down at 37°C for 1 min. The fluorescence signals from HEX and FAM for the specific alleles were detected using the same Detection System (Bio-Rad, Hercules, CA, USA) at 37°C after thermal cycling was complete. The physical position of the KASP markers was determined by blasting primers against the barley reference genome sequences (Mascher et al., 2017, 2021; Monat et al., 2019) using blastN at the IPK barley blast server (https://galaxy-web.ipk-gatersleben.de).

### Linkage Analysis

Linkage analysis was performed by setting the number of recombinant gametes in relation to the number of gametes analyzed (Pellio et al., 2005). The genetic resolution of the population (% recombination) was calculated by dividing the number 1 by the number of gametes. To correct for those plants which died during cultivation, a “Corrected genetic resolution” for the remaining RILs was applied by dividing the % recombination identified for the F_2_ generation by the number of those remaining RILs (Lüpken et al., 2013).

### Collinearity of the Target Region Between Resistant and Susceptible Cultivars

The physical position of the new flanking markers identified in the present study was determined according to the sequence assembly of Morex v3. In order to visually compare the target region between the genotypes Chikurin Ibaraki 1, Igri, and Morex (Jayakodi et al., 2020; Mascher, 2020), the flanking markers were blasted against the whole-genome sequence of Chikurin Ibaraki 1 and Igri by using the tool Multiple Alignment using Fast Fourier Transform (MAFFT; Katoh and Standley, 2013) in the Galaxy web server (Giardine et al., 2005; Blankenberg et al., 2010; Goecks et al., 2010). The target region was identified in these two genotypes according to the best hits of both flanking markers, and the alignments of the target region between the three genotypes were plotted and visualized as a dot-plot with D-GENIES webpage (Cabanettes and Klopp, 2018) by using the Minimap2 aligner (Li, 2018).

### Identification and Re-Sequencing of Candidate Genes

In the target region, the HC and LC genes were identified according to the gene annotation of Morex v3 (Mascher, 2020).^1^ In order to extract the corresponding genes from Chikurin Ibaraki 1 assembly data, the sequences of HC and LC genes in the target interval of Morex were used as query for a BLASTN (Altschul et al., 1997) search against the target region of Chikurin Ibaraki 1. For the susceptible parental line Igri, annotated genes in the target interval were identified according to the pan-genome database available on the IPK Galaxy Blast Suite (Deng et al., 2007; Jayakodi et al., 2020).^2^

In order to obtain the gene sequence of 6 HC and 2 LC genes in the second susceptible parental line Uschi, based on the gene sequences of Morex v3 and Igri, the corresponding primers for re-sequencing of all identified genes were developed by using the online tool primer3 (Supplementary Table S2).^3^ PCR amplification was conducted in a 30 μl reaction volume consisting of 3 μl of template DNA (25-30 ng/μl), 3 μl of 10 × buffer BD (detergent-free buffer), 3 μl of 25 mM MgCl_2_, 0.6 μl of 10 mM dNTP-Mix, 0.75 μl of each forward primer (10.0 pmol/μl) and reverse primer (10.0 pmol/μl), 0.6 μl of HOT FIREPol DNA polymerase (Solis BioDyne, Tartu, Estonia) and 18.3 μl double distilled water. The DNA was amplified in a GeneAmp PCR System 9,700 (Applied Biosystems) under the following conditions: 94°C for 5 min; followed by touchdown PCR with 12 cycles of 30 s at 94°C, 30 s at 62°C, 30 s at 72°C; and then 35 cycles with 30 s at 94°C, 30 s at 56°C, 30 s at 72°C; and a final extension at 72°C for 10 min. Amplified products (1 μl) were checked on an agarose gel (1.5%) and analyzed using the imaging system Gel Doce™ XR and the Quantity One® 1-D analysis software (4.6.2; Bio-Rad, Hercules, CA, USA). PCR products were purified and sequenced by the company Microsynth AG (Balgach, Switzerland). Obtained sequences were edited and the polymorphisms between parental lines (Chikurin Ibaraki 1, Igri, and Uschi) were identified using Sequencher 5.1 software (Gene Codes, Ann Arbor, MI, United States).

## Results

### High-Resolution Mapping Populations for *rym15*

Two crosses were used for the construction of the high-resolution mapping populations. In total, 2,218 and 5,870 F_2_ plants derived from I × C and C × U were sown, of which 2,174 and 5,728 germinated and were analyzed subsequently. From these, 162 (3.725% recombination) and 288 (2.514% recombination) segmental recombinant F_2_ plants were identified, respectively (Table 1). Initially, for the population I × C, a total of 2,174 F_2_ plants providing a genetic resolution of 0.0230% recombination was screened for recombination events between the previous flanking markers *rym15*_1 and *rym15*_8 and a genetic distance of 3.725% recombination was determined. Due to the non-survival of recombinant plants, the corrected genetic resolution provided by 139 remaining RILs equaled 0.02679% recombination. For population C × U, a total of 5,728 F_2_ plants providing a genetic resolution of 0.0087% recombination were screened for recombination events between the flanking markers *rym15*_1 and *rym15*_8 and a genetic distance of 2.514% recombination was determined. Due to the non-survival of recombinant plants, the corrected genetic resolution provided by 284 remaining RILs equaled 0.00885% recombination.

### BaMMV Phenotyping

The BaMMV infection experiment showed a segregation of 67 resistant and 72 susceptible, as well as 140 resistant and 144 susceptible RILs in the population I × C and C × U, respectively, which fit to the expected 1r:1 s ratio. Chi-square test in the population I × C (*χ*^2^ 1r:1 s = 0.180, df = 1, *p* = 0.6714) and C × U (*χ*^2^ 1r:1 s = 0.056, df = 1, *p* = 0.8129) for goodness of fit indicated that the resistance against BaMMV is controlled by a single gene (*rym15*) in both populations (Table 1).

### Marker Saturation of the *rym15* Locus

GBS analysis of three parental lines identified 27,017 (Chikurin Ibaraki 1 and Igri) and 29,197 (Chikurin Ibaraki 1 and Uschi) polymorphisms. In total, 20,099 polymorphisms (74.39%) were identical among both comparisons. On the target chromosome 6H, a set of 3,388 (Chikurin Ibaraki 1 and Igri) and 3,813 (Chikurin Ibaraki 1 and Uschi) polymorphisms was identified, of which 2,488 (73.44%) were in common. In the target region between the previous flanking markers *rym15*_1 and *rym15*_8, a set of 365 (Chikurin Ibaraki 1 and Igri) and 396 (Chikurin Ibaraki 1 and Uschi) polymorphisms was identified, of which 301 (82.47%) were in common (Supplementary Table S3).

**Figure 1:**
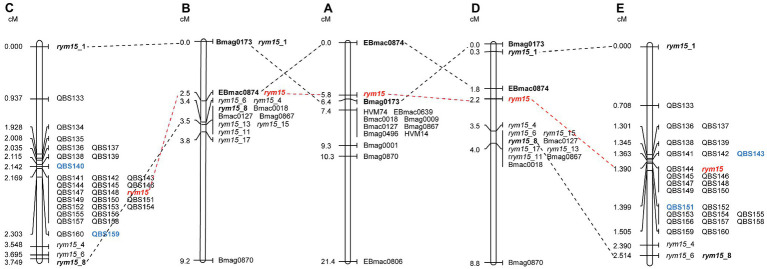
Genetic maps of *rym15* and collinearity of common molecular markers. **(A)** An initial genetic map of *rym15* based on a set of 217 DH lines derived from the cross of Chikurin Ibaraki 1 × Plaisant (Le Gouis et al., 2004). **(B)** Medium-resolution map of *rym15* based on a set of 180 F_2_ lines derived from the population Igri×Chikurin Ibaraki 1 (Wang et al., 2021). **(C)** High-resolution map of *rym15* based on a set of 139 F_4_ segmental RILs in the population Igri ×Chikurin Ibaraki 1. **(D)** Medium-resolution map of *rym15* based on a set of 342 F_2_ lines derived from the population Chikurin Ibaraki 1 × Uschi (Wang et al., 2021). **(E)** High-resolution map of *rym15* based on a set of 284 F_4_ segmental RILs in the population Chikurin Ibaraki 1 × Uschi. The target gene *rym15* is highlighted in red, the bold font indicates previous flanking markers from the initial and medium-resolution maps, while the new flanking markers identified from high-resolution mapping are shown in blue.

The *rym15* target region was saturated with a set of 32 KASP markers that span a 133 Mb interval on chromosome 6H in Morex v3. Out of these 32 markers, three polymorphisms (QBS134, QBS135, and QBS140) could not be reproduced in the population C × U (Supplementary Table S1). In the population I × C, mapping of all 32 markers reduced the target interval of *rym15* from 3.5 cM to a smaller region of 0.161 cM between markers QBS140 and QBS159, and 18 markers co-segregated with the target locus (Figure 1). In the population C × U, analysis of all 29 markers reduced the interval harboring *rym15* from 3.7 cM to 0.036 cM between markers QBS143 and QBS151, and 7 markers co-segregated with the target gene *rym15* (Figure 1).

**Figure 2:**
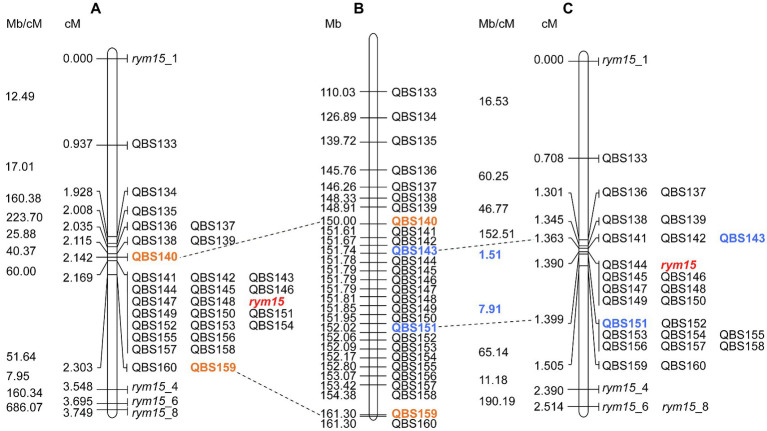
High-resolution genetic maps of *rym15* and physical map of barley chromosome 6HS. **(A)** High-resolution map of *rym15* based on a set of 139 F_4_ RILs in the population Igri×Chikurin Ibaraki 1. **(B)** Physical map of Morex on chromosome 6HS according to Morex v3. **(C)** High-resolution map of *rym15* based on a set of 284 F_4_ RILs in the population Chikurin Ibaraki 1 × Uschi. The recombination rates (Mb/cM) are listed left of the genetic maps for both populations. The target gene *rym15* is highlighted in red and the flanking markers are shown in orange (Igri×Chikurin Ibaraki 1) and blue (Chikurin Ibaraki 1 × Uschi). Crucial recombination events in the population Chikurin Ibaraki 1 × Uschi are highlighted in blue.

BLASTN comparison of marker sequences against the barley reference sequence Morex v3 revealed that all mapped markers are co-linear genetically and physically in both mapping populations, and the physical size of the target region in the population I × C and C × U is 11.3 Mb and 0.28 Mb, respectively (Figure 2). The marker saturation revealed a large difference of recombination distribution between the two populations (Figure 2). In the population C × U, the recombination frequencies have been estimated from 1.51 to 190.19 Mb/cM, while the population I × C shows suppressed recombination, of which the physical/genetic ratio varies from 7.95 to 686.07 Mb/cM. In the population C × U, the recombination event between markers QBS143 and QBS144 (1.51 Mb/cM), as well as QBS150 and QBS151 (7.91 Mb/cM) are crucial for mapping the target gene *rym15* to a smaller interval of 0.28 Mb. In contrast, those markers co-segregated with *rym15* in the population I × C (Figure 2).

### Similarity of Target Region Between Parental Lines

Taking advantage of the second population C × U, the physical size of the target region between markers QBS143 and QBS151 encompassed 281 kb according to Morex v3 (Figure 2). Blasting the flanking marker sequences against the Chikurin Ibaraki 1 and Igri genome assemblies revealed that the corresponding physical size of the target region is around 282 and 285 kb, respectively (Supplementary Figure S1). A dot-plot analysis comparing the target region between the parental lines Chikurin Ibaraki 1 and Igri, and between Chikurin Ibaraki 1 and Morex v3, revealed a substantial co-linearity and similarity with identity ranging from 75 to 100% (Supplementary Figure S1). The micro co-linearity between physical and genetic order of all used markers was consistent. In the other pan-genome accessions, the physical size of the target region ranged from 0.26 (Golden Promise) to 0.34 Mb (HOR3365; Supplementary Table S4).

### Candidate Gene Analysis at the *rym15* Locus

**Figure 3:**
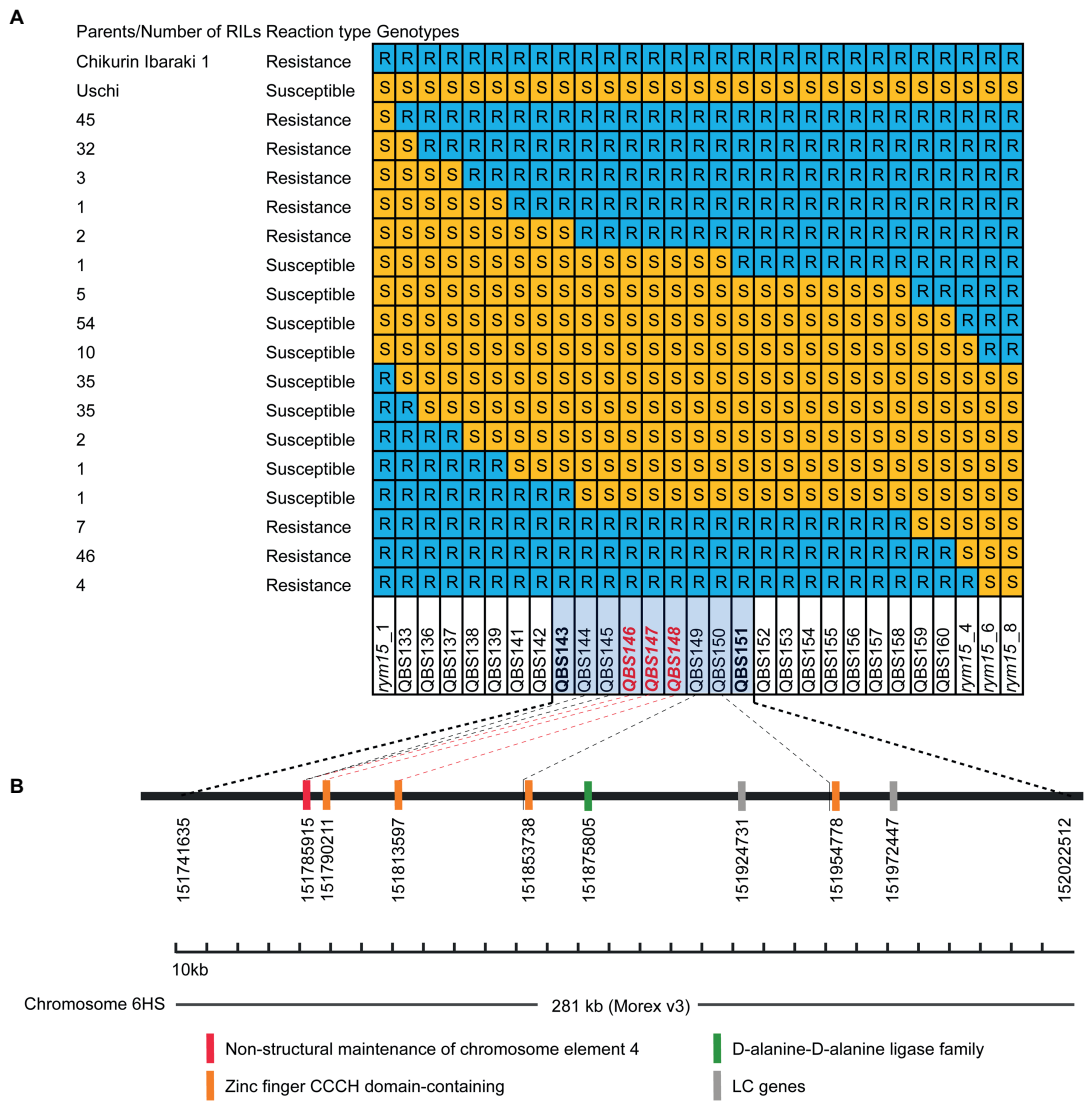
Candidate genes in the *rym15* target region of 281 kb. **(A)** Graphical genotypes of 284 F_4_ RILs derived from population Chikurin Ibaraki 1 × Uschi carrying recombination events between *rym15*_1 and *rym15*_8. Marked with red color are those located in the coding region of high confidence genes. **(B)** A set of six high confidence and two low confidence genes positioned in the target interval.

In our previous medium-resolution maps of *rym15*, the interval was mapped between the two markers *rym15*_1 and *rym15*_8 with a physical size of 133 Mb according to the Morex v3 reference genome. In this region, 620 HC genes and 1,025 LC genes are located (Wang et al., 2021). Due to extensive marker saturation in the present study, the physical size of the target region was reduced to 281 kb in the population C × U. In this region a set of 8 genes was identified, of which 6 are HC and two are LC genes. The annotation of two LC genes HORVU.MOREX.r3.6HG0573640 and HORVU.MOREX.r3.6HG0573660 are ATP-dependent DNA helicase and Retrovirus-related Pol polyprotein from transposon TNT 1–94, respectively. Out of the 6 HC genes, four encode zinc finger CCCH domain-containing proteins (HORVU.MOREX.r3.6HG0573600, HORVU.MOREX.r3.6HG0573610, HORVU.MOREX.r3.6HG0573620 and HORVU.MOREX.r3.6HG0573650). The other two HC genes are coding for non-structural maintenance of chromosome element 4 (NSE4) and D-alanine-D-alanine ligase family (HORVU.MOREX.r3.6HG0573590 and HORVU.MOREX.r3.6HG0573630; Figure 3). Meanwhile, according to the annotation data of Igri, in the target region, the same number of the HC genes was found with the same order and description as in Morex v3 (Horvu_IGRI_6H01G211100.1, Horvu_IGRI_6H01G211200.1, Horvu_IGRI_6H01G211300.1, Horvu_IGRI_6H01G211400.1, Horvu_IGRI_6H01G211500.1, and Horvu_IGRI_6H01G211600.1). Furthermore, the order of those 6 HC and two LC genes in Chikurin Ibaraki 1 was revealed to be the same as in Morex and Igri. Finally, the alignment analysis of the coding region of the 6 HC and two LC genes from three parental lines shows that three HC genes (HORVU.MOREX.r3.6HG0573620, HORVU.MOREX.r3.6HG0573630, and HORVU.MOREX.r3.6HG0573650) and two LC genes (HORVU.MOREX.r3.6HG0573640 and HORVU.MOREX.r3.6HG0573660) are monomorphic between resistant and susceptible genotypes. In contrast, for the remaining three HC genes, one functional SNP was identified for each of the genes (HORVU.MOREX.r3.6HG0573590, HORVU.MOREX.r3.6HG0573600, and HORVU.MOREX.r3.6HG0573610; Table 2).

**Table 2:** Functional SNPs between resistant (Chikurin Ibaraki 1) and susceptible (Igri, Uschi and Golden Promise) lines originated from three candidate genes.

Gene			SNP				Codon	Amino acid substitution
Morex v3	Igri	Golden Promise	Chikurin Ibaraki 1	Igri	Uschi	Golden Promise		
HORVU.MOREX.r3.6HG0573590.1	Horvu_IGRI_6H01G211100.1	Horvu_GOLDEN_6H01G188600.1	G	T	T	T	GCA- > TCA	Ala(A)- > Ser(S)
HORVU.MOREX.r3.6HG0573600.1	Horvu_IGRI_6H01G211200.1	Horvu_GOLDEN_6H01G188700.1	G	A	A	G	GAC- > AAC	Asp(D)- > Asn(N)
HORVU.MOREX.r3.6HG0573610.1	Horvu_IGRI_6H01G211300.1	Horvu_GOLDEN_6H01G188800.1	A	G	G	G	TGA- > TGG	Ter(*)- > Trp(W)

Further analyses of the sequence of Golden Promise, which is susceptible to BaMMV, revealed the same three HC genes (Horvu_GOLDEN_6H01G188600, Horvu_GOLDEN_6H01G188700, and Horvu_GOLDEN_6H01G188800). The alignment of these three HC genes between Chikurin Ibaraki 1 and Golden Promise revealed that one HC gene (Horvu_GOLDEN_6H01G1887000) has the same coding sequence in both genotypes. For the remaining two HC genes Horvu_GOLDEN_6H01G188600 and Horvu_GOLDEN_6H01G188800, one functional SNP was detected in each gene between Chikurin Ibaraki 1 and Golden Promise. Thus, only two HC genes (HORVU.MOREX.r3.6HG0573590 and HORVU.MOREX.r3.6HG0573610) are promising candidates in the target region (Table 2). Meanwhile, it was shown that the functional SNPs-derived KASP markers QBS146 (located in HC gene HORVU.MOREX.r3.6HG0573590) and QBS148 (located in HC gene HORVU.MOREX.r3.6HG0573610) co-segregated with the target locus *rym15* in both populations.

## Discussion

In the present study, phenotypic analysis of 423 F_4_ segmental RILs showed that the BaMMV resistance of Chikurin Ibaraki 1 is controlled by a single gene. This confirms results of previous studies (Le Gouis et al., 2004; Wang et al., 2021). By high-resolution mapping, the target region harboring *rym15* was narrowed down to 281 kb and 6 HC candidate genes were identified for the BaMMV resistance locus *rym15*. Functional SNPs between resistant and susceptible genotypes were detected in only two HC genes, representing a substantial step toward cloning of *rym15*.

It is well known that recombination rates are not fixed and a significant inter-individual variability has been reported for virtually every species, such as bacteria, fungi, plants, and animals (Simchen and Stamberg, 1969; Brooks, 1988; Fisher-Lindahl, 1991; Petes et al., 1991). Various studies about recombination rates and gene densities in barley show that gene density is not uniform along the chromosome and is usually correlated with recombination frequency (Han et al., 1998; Künzel et al., 2000; Rostoks et al., 2002). On chromosome 6HS, the calculated recombination frequency and gene density are not high in the region between markers *rym15*_1 and *rym15*_8 (Muñoz-Amatriaín et al., 2015). In the present study, the use of two different mapping populations reflects the different recombination rates within a defined interval. The population I × C showed a reduced recombination rate in this region compared with the population C × U. A set of 18 and 7 markers co-segregated with the target locus *rym15* in the population I × C and C × U, respectively. Four markers, which co-segregated with *rym15* in the population I × C revealed crucial recombination events between QBS143 and QBS144 (1.51 Mb/cM), as well as QBS150 and QBS151 (7.91 Mb/cM) in the population C × U, facilitating narrowing of the *rym15* interval to 281 kb.

The accuracy of genome sequence information in the target region is key to identifying candidate genes in a resistance donor. Previously, cloning of BaYMV/BaMMV recessive resistance genes *rym4*/*5* and *rym1*/*11* was assisted by bacterial artificial chromosome (BAC) clones, which is a cumbersome and time-consuming process (Stein et al., 2005; Yang et al., 2014). As third-generation sequencing technologies recently become achievable and affordable, a recent study comparing different long-read sequencing methods revealed that the PacBio HiFi sequencing method performed best for sequence assembly of barley (Mascher et al., 2021). In the present study, re-sequencing of the resistant donor Chikurin Ibaraki 1 was conducted using PacBio HiFi reads. Finally, a set of two HC genes was identified with the assistance of the whole-genome assembly of Chikurin Ibaraki 1. In future, this assembly may be used to map another recessive BaYMV resistance gene present in Chikurin Ibaraki 1, which is located on chromosome 5HS (Werner et al., 2003). The availability of the barley pan-genome, comprising a set of 20 diverse barley accessions including the population I × C susceptible parental line Igri (Jayakodi et al., 2020), was critically important for the *rym15* candidate gene identification.

It is well known that new pathogen variants may be virulent to major resistance genes. For example, the isolated resistance gene *rym4*/*5* has been overcome in different regions of Europe and East Asia, and another resistance gene *rym1*/*11* became susceptible to isolate BaYMV-CN_NY in China as well (Kühne et al., 2003; Kanyuka et al., 2004; Habekuß et al., 2007; Nishigawa et al., 2008; Rolland et al., 2017; Jiang et al., 2022). These examples highlight the importance of identifying new genetic resources that are resistant to new virulent virus isolates. The two HC genes carrying functional SNPs between resistant and susceptible cultivars are NSE4 (HORVU.MOREX.r3.6HG0573590) and a zinc finger CCCH domain-containing protein (HORVU.MOREX.r3.6HG0573610), which have not yet been reported as resistance genes against BaMMV/BaYMV. According to the information obtained from UniProt (https://www.uniprot.org/), the candidate gene HORVU.MOREX.r3.6HG0573590 promotes sister chromatid alignment after DNA damage and facilitates double-stranded DNA break (DSBs) repair *via* homologous recombination between sister chromatids (Watanabe et al., 2009). In contrast, the other candidate gene HORVU.MOREX.r3.6HG0573610 encodes a zinc finger CCCH domain-containing protein. This kind of protein was reported to be involved in cell fate specification and developmental processes in plants, as well as in the response to biotic and abiotic stress (Ai et al., 2022). Several studies confirmed that the CCCH-type zinc finger protein is responsible for resistance against different pathogens in different plant species. For example, a novel CCCH-type zinc finger protein GhZFP1 derived from cotton (*Gossypium hirsutum*) positively regulates resistance to the fungal pathogen *Rhizoctonia solani* in tobacco (Guo et al., 2009). The study of rice CCCH-type zinc finger protein C3H12 concluded that this gene is positively regulated to mediate resistance against the bacterial pathogen *Xoo* (Deng et al., 2012). Another study shows that the pepper TZnF protein CaC3H14 is involved in the defense response of pepper to infection by *Ralstonia solanacearum* (Qiu et al., 2018). Furthermore, an Arabidopsis CCCH protein C3H14 is a positive regulator for basal defense against *Botrytis cinerea* mainly by WRKY33 signaling (Wang et al., 2020). Moreover, the predicted K homology (KH) domain in the gene HORVU.MOREX.r3.6HG0573610 usually has an RNA-binding function (Burd and Dreyfuss, 1994). Considering all the evidence, it seems that the gene HORVU.MOREX.r3.6HG0573610 is the most likely candidate for BaMMV resistance encoded by *rym15*. Functional analysis of the two candidate genes, for example by gene editing (Hoffie et al., 2021) will likely lead to cloning of the causal gene for *rym15*.

## Conclusion

In the present study, two high-resolution mapping populations were constructed, comprising 423 F_4_ segmental RILs from the crosses of I × C (139 RILs) and C × U (284 RILs). Phenotypic analysis revealed that the resistance against BaMMV encoded by *rym15* is controlled by a single gene. Using combinations of different whole-genome and targeted sequencing methods, detected polymorphisms between parental lines were converted to KASP markers and subsequently analyzed on all RILs. Combining the genetic and phenotypic data, two high-resolution maps were constructed. The physical size of the target region was reduced to a 0.28 Mb region containing six HC and two LC genes. Taking advantage of public genome assemblies including the susceptible cultivar Golden Promise and Igri assembly data, functional SNPs between resistant and susceptible parental lines were detected in only two HC genes. However, the functional analysis of these two genes is still needed to identify the causal gene for *rym15*.

## Data Availability Statement

The datasets presented in this study can be found in online repositories. The names of the repository/repositories and accession number(s) can be found at: NCBI with accession PRJEB50079.

## Author Contributions

DP and FO conceived the project, acquired the funding, and designed the experiments. AH provided the initial F_2_ populations. YW performed the experiments and wrote the manuscript. JF carried out the re-sequencing of Chikurin Ibaraki 1. YW and DP analyzed the data. MJ and MM conducted the genome assembly of Chikurin Ibaraki 1. FO, RS, AS, and DP edited the manuscript. All authors contributed to the article and approved the submitted version.

## Funding

The research was funded in frame of the IdeMoDeResBar project [FKZ 031B0199 (phase 1) and 031B0887 (phase 2)] by the German Federal Ministry of Education and Science (BMBF). The sequencing of Chikurin Ibaraki 1 was partially supported by the DFG Research Infrastructure NGS_CC (project 407495230) as part of the Next Generation Sequencing Competence Network (project 423957469).

## Conflict of Interest

The authors declare that the research was conducted in the absence of any commercial or financial relationships that could be construed as a potential conflict of interest.

## Publisher’s Note

All claims expressed in this article are solely those of the authors and do not necessarily represent those of their affiliated organizations, or those of the publisher, the editors and the reviewers. Any product that may be evaluated in this article, or claim that may be made by its manufacturer, is not guaranteed or endorsed by the publisher.

## Footnotes

^1^ https://wheat.pw.usda.gov/GG3/content/morex-v3-files-2021

^2^ https://galaxy-web.ipk-gatersleben.de/

^3^ http://primer3.ut.ee/cgi-bin/primer3/primer3web_results.cgi

## Supplementary Material

Supplementary Figure S1 Collinearity of **(A)** Chikurin Ibaraki 1 vs. Morex v3 and **(B)** Chikurin Ibaraki 1 vs. Igri genome assemblies in the target region between markers QBS143 and QBS151. Colored boxes with red and green indicate high confidence and low confidence genes, respectively.
