# Supplementary figures and images for: High-Resolution Mapping of Barley mild mosaic virus Resistance Gene rym15
Source: Front Plant Sci. 2022 Jun 2;13:908170. doi: 10.3389/fpls.2022.908170 (PMC9201720; doi:10.3389/fpls.2022.908170)

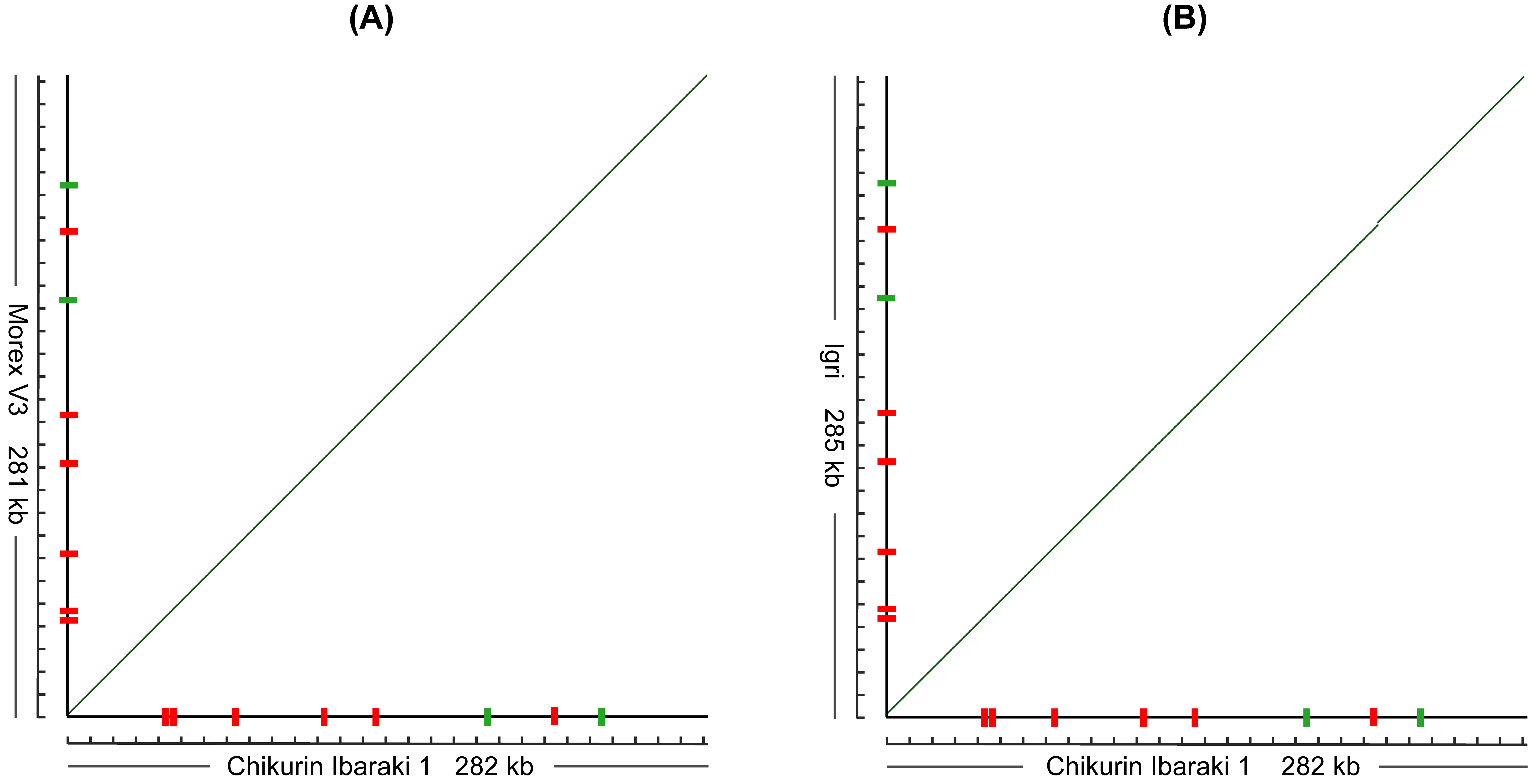

Supplement: Supplementary Figure S1 — Collinearity of (A) Chikurin Ibaraki 1 vs. Morex v3 and (B) Chikurin Ibaraki 1 vs. Igri genome assemblies in the target region between markers QBS143 and QBS151. Colored boxes with red and green indicate high confidence and low confidence genes, respectively. [file Image_1.TIF]
